# Salvianolic Acid B Preserves Myocardial Viability and Extends the Preservation Window of Mouse Hearts During Static Cold Storage

**DOI:** 10.3390/ph19071082

**Published:** 2026-07-14

**Authors:** Junyi Wang, Han Wang, Guangyi Cui, Chao Lin

**Affiliations:** School of Medicine, Nanjing University of Chinese Medicine, 138 Xianlin Avenue, Nanjing 210023, China

**Keywords:** organ preservation, autophagy, cardioprotection, University of Wisconsin solution, salvianolic acid B, ischemia–reperfusion injury

## Abstract

**Background:** Heart transplantation remains the definitive therapy for end-stage heart failure, but its success critically depends on optimal donor heart preservation. Although the University of Wisconsin (UW) solution is the clinical gold standard, its safe storage window for hearts is limited to 4–6 h. Extending this static cold storage (SCS) window represents a critical challenge. We hypothesized that Salvianolic acid B (SalB), a primary bioactive compound in Salvia miltiorrhiza with potent antioxidant and cardioprotective properties, could serve as an effective pharmacological additive to UW solution. **Methods:** Isolated mouse hearts were subjected to SCS at 4 °C for varying durations (4, 8, and 10 h) in either standard UW solution or UW solution supplemented with 0.8 mg/mL SalB. Myocardial injury was assessed via cardiac enzyme leakage (BNP, CK-MB, LDH), oxidative stress markers, sterile inflammation, and histopathology. The regulatory roles of SalB on apoptosis and autophagic flux were further evaluated, utilizing the autophagy inhibitor 3-methyladenine (3-MA) to establish mechanistic causality. **Results:** Hearts preserved in SalB-supplemented UW solution exhibited significantly ameliorated histopathological damage, reduced cold ischemia-induced enzyme efflux, and suppressed oxidative stress compared to standard UW solution. Notably, hearts preserved for 8 h with SalB maintained structural and biochemical integrity comparable to those stored for only 4 h in standard UW solution. Mechanistic investigations revealed that SalB treatment orchestrates a pro-survival shift by significantly reducing cardiomyocyte apoptosis and robustly enhancing autophagic flux to maintain cellular homeostasis. The abrogation of autophagy by 3-MA effectively reversed these cardioprotective benefits. **Conclusions:** Supplementing UW solution with SalB effectively prolongs the structural and biochemical preservation of donor hearts during cold storage up to 8–10 h. By mitigating ischemia–reperfusion injury via the targeted modulation of autophagic flux, SalB highlights its strong translational potential as a robust pharmacological additive for heart transplantation.

## 1. Introduction

Optimal organ preservation is a critical determinant of successful transplantation outcomes following revascularization. For patients with end-stage organ failure, transplantation often represents the sole life-saving intervention. However, due to the severe shortage of donor organs, only a limited number of patients receive this therapy. Therefore, maximizing the viability and utilization of available grafts through superior preservation strategies is paramount. During transplantation, the graft is inevitably exposed to prolonged ischemia and hypoxia. The consequent energy depletion and shift to anaerobic metabolism lead to a rapid accumulation of toxic metabolites, which subsequently triggers sterile inflammation and profound tissue injury [1,2].

In 1969, Collins et al. pioneered the preservation of canine kidneys for 12 h using a chilled salt solution, laying the foundation for modern static cold storage (SCS) [3,4]. Building on these foundational advances, current donor organ preservation strategies are primarily categorized into SCS, hypothermic machine perfusion (HMP), and normothermic machine perfusion (NMP). While machine perfusion techniques are gaining clinical traction, SCS remains the predominant method worldwide due to its cost-effectiveness, logistical simplicity, and extensive clinical validation [5,6]. In SCS, the donor organ is flushed to remove blood and submerged in a hypothermic preservation solution. This hypothermia suppresses metabolic demand, thereby increasing ischemic tolerance. However, prolonged hypothermic storage inherently induces cold ischemic injury, compromising graft function and exacerbating subsequent ischemia–reperfusion injury (IRI) upon reperfusion.

The UW solution, developed by Belzer et al., is currently the most widely utilized organ preservation medium in clinical practice [7] and serves as the gold standard for preserving the kidney, liver, and intestine [8]. Nevertheless, preservation in standard UW solution may still result in suboptimal functional recovery and carries a high risk of ischemia-related complications, such as biliary tract damage [9]. Consequently, extensive research has focused on optimizing preservation outcomes by incorporating targeted pharmacological additives into the preservation solution. Such optimizations aim to safely prolong the ischemic interval, improve graft utilization, and enhance postoperative survival [10,11].

*Salvia miltiorrhiza* is a well-established traditional Chinese medicine (TCM) renowned for its efficacy in promoting microcirculation. Its primary water-soluble and lipid-soluble constituents are salvianolic acids and tanshinones, respectively. Previous studies have demonstrated the protective properties of *S. miltiorrhiza* across various preserved organs, including the liver, heart, intestine, and kidney. Notably, SalB has proven highly effective in mitigating cold preservation-induced graft damage [12,13,14,15,16,17,18]. Furthermore, autophagy—a highly conserved catabolic process responsible for degrading and recycling damaged cytoplasmic components—has emerged as a critical adaptive mechanism against myocardial ischemic stress. Evidence suggests that SalB mitigates myocardial injury by modulating autophagic flux, thereby preserving cell viability, inhibiting apoptosis, and maintaining intracellular homeostasis [19]. Therefore, the objective of this study was to evaluate the cardioprotective efficacy of SalB as an additive to UW solution during the prolonged cold storage of isolated mouse hearts, and to elucidate the underlying autophagic mechanisms.

## 2. Results

### 2.1. Cardioprotective Efficacy of SalB During Cold Storage of Mouse Hearts

#### 2.1.1. SalB Attenuates Myocardial Enzyme Leakage

**Figure 1:**
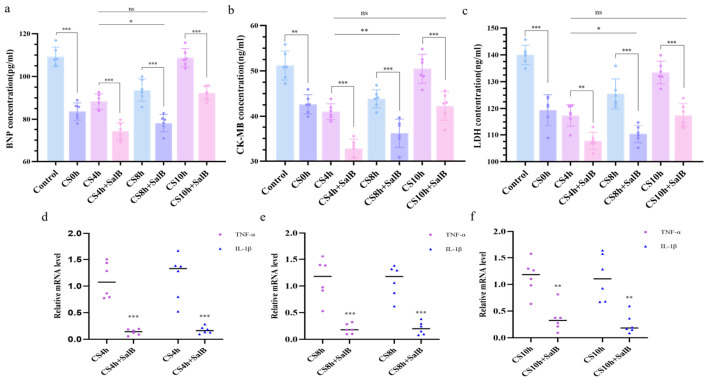
SalB mitigates cold ischemia-induced injury and bolsters antioxidant capacity in transplanted mouse hearts. (**a**–**c**) Quantification of cardiac enzyme leakage (BNP, CK-MB, and LDH) in the coronary effluent via ELISA (n = 6). (**d**–**f**) RT-qPCR analysis of myocardial pro-inflammatory cytokine mRNA expression (*IL-1β* and *TNF-α*) following 4 h, 8 h, and 10 h of cold storage (n = 6). Statistical significance was determined using one-way ANOVA followed by Tamhane’s T2 post hoc test. ns, relatively not significant (*p* > 0.05); * *p* < 0.05; ** *p* < 0.01; *** *p* < 0.001.

Myocardial enzymes are critical biomarkers for evaluating the severity of cardiomyocyte injury [20,21]. While the CS0h group exhibited minimal enzyme leakage, cold storage for 4, 8, and 10 h induced a time-dependent increase in the control groups. However, the levels of BNP, CK-MB, and LDH in the SalB-treated groups (CS4h+SalB, CS8h+SalB, and CS10h+SalB) were markedly lower than those in their respective untreated counterparts (CS4h, CS8h, and CS10h). Notably, hearts preserved for 8 h with SalB exhibited a substantial reduction in enzyme leakage, maintaining levels comparable to those of the 4 h untreated group, whereas this compensatory advantage was no longer significant at 10 h (Figure 1a–c).

#### 2.1.2. SalB Mitigates Oxidative Stress and Preserves Antioxidant Capacity

**Figure 2:**
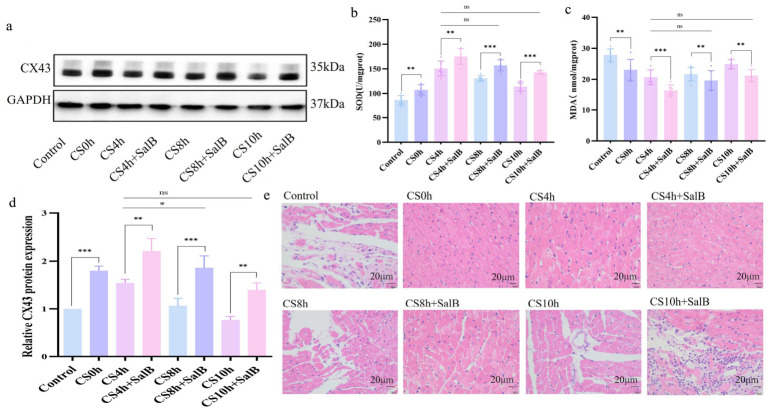
(**a**) Representative Western blots and (**d**) quantitative analysis of CX43 protein expression in myocardial tissues (n = 3). (**b**,**c**) Assessment of myocardial antioxidant capacity based on SOD activity and MDA content (n = 6). (**e**) Representative H&E staining images demonstrating myocardial tissue architecture across groups, scale bar = 20 μm (original magnification, 400×). Data are presented as mean ± SD. Statistical significance was determined using one-way ANOVA followed by Tamhane’s T2 post hoc test. ns, relatively not significant (*p* > 0.05); * *p* < 0.05; ** *p* < 0.01; *** *p* < 0.001.

The antioxidant defense of myocardial tissues was significantly bolstered by the addition of SalB to the preservation solution. Specifically, while baseline hearts (CS0h) exhibited high SOD activity and low MDA accumulation, prolonged cold storage severely impaired this balance. Supplementation of the UW solution with SalB effectively rescued SOD activity and markedly suppressed MDA generation. Crucially, in comparison to the 4 h standard cold storage group, the 8 h and 10 h SalB-treated groups demonstrated no significant deterioration in their antioxidant profiles (SOD or MDA levels), indicating robust and sustained protection against oxidative damage (Figure 2b,c).

#### 2.1.3. SalB Downregulates Pro-Inflammatory Cytokine Expression

Sterile inflammation is a pivotal driver of myocardial ischemic injury, with *IL-1β* and *TNF-α* serving as representative markers [22,23]. (Figure 1d–f).

#### 2.1.4. SalB Preserves Connexin43 (CX43) Integrity

CX43 is essential for maintaining gap junction communication, and its degradation in cardiomyocytes is closely associated with ischemic cardiomyopathy and clinical congestive heart failure [24]. Western blot analysis demonstrated that SalB treatment protected CX43 protein from cold ischemia-induced degradation. Compared to the baseline CS0h group, prolonged storage decreased CX43 levels; however, CX43 expression was significantly rescued in all SalB-treated groups (CS4h+SalB, CS8h+SalB, and CS10h+SalB) compared to their untreated counterparts. Furthermore, CX43 expression in the 8 h SalB-treated group remained significantly elevated relative to the 4 h untreated group, with no significant difference detected between the 10 h SalB-treated and 4 h untreated hearts (Figure 2a,d).

#### 2.1.5. SalB Ameliorates Histopathological Damage

Histological evaluation revealed a time-dependent exacerbation of structural injury in standard UW groups compared to the baseline CS0h group. Conversely, tissue architecture, including interstitial edema and myofiber arrangement, was substantially preserved and ameliorated in the SalB-supplemented groups (CS4h+SalB, CS8h+SalB, and CS10h+SalB) relative to their time-matched standard controls (Figure 2e).

### 2.2. Mechanisms Underlying SalB-Mediated Cardioprotection

#### 2.2.1. SalB Suppresses Cardiomyocyte Apoptosis

**Figure 3:**
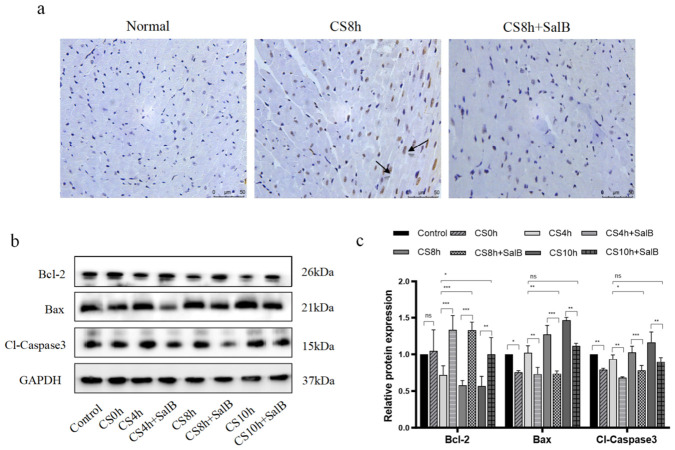
SalB exerts an anti-apoptotic effect during prolonged cold storage. (**a**) Representative TUNEL staining images assessing cardiomyocyte apoptosis. Brown nuclei indicate apoptotic cells, while blue-purple indicates normal cell nuclei. Scale bar = 50 μm (original magnification, 400×). (**b**) Representative Western blots and (**c**) corresponding quantitative analysis of apoptosis-related proteins (Bcl-2, Bax, and Cleaved Caspase-3) in myocardial tissues (n = 3). Data are presented as mean ± SD. ns, relatively not significant (*p* > 0.05); * *p* < 0.05; ** *p* < 0.01; *** *p* < 0.001 vs. respective groups.

TUNEL staining demonstrated a pronounced increase in apoptotic nuclei (indicated by brown-stained areas) in the CS8h group relative to normal tissues. This apoptotic response was notably blunted in the CS8h+SalB group (Figure 3a). At the molecular level, baseline hearts (CS0h) exhibited elevated Bcl-2 expression alongside diminished Bax and Cleaved Caspase-3 levels compared to controls. Following cold storage, SalB induced a distinct pro-survival shift across all time points, characterized by upregulated anti-apoptotic Bcl-2 expression and downregulated pro-apoptotic Bax and Cleaved Caspase-3 levels, relative to the untreated groups. Importantly, the anti-apoptotic profile of the 8 h SalB group was significantly superior to that of the 4 h untreated group, though this advantage equalized by the 10 h mark (Figure 3b,c).

#### 2.2.2. SalB Enhances Autophagic Flux in Cold-Stored Mouse Hearts

**Figure 4:**
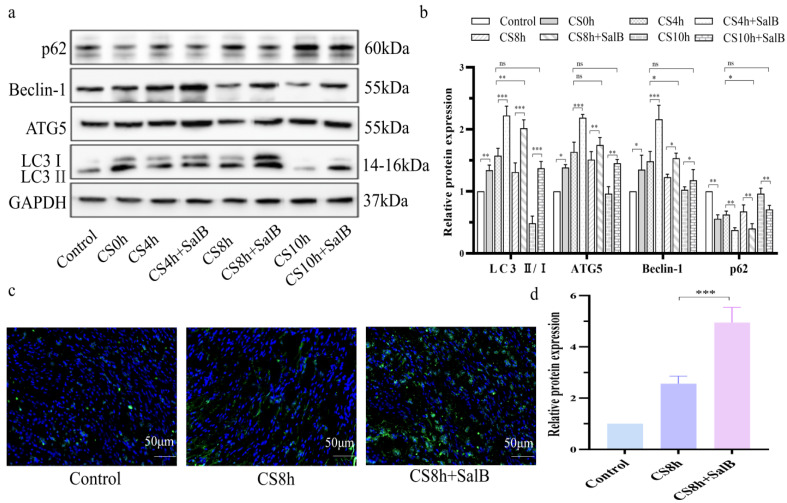
SalB enhances autophagic flux in cold-stored mouse hearts. (**a**) Representative Western blots and (**b**) quantitative analysis of autophagy markers (LC3 II/I, p62, ATG5, and Beclin-1) (n = 3). (**c**) Representative immunofluorescence images of LC3 expression (green) with DAPI nuclear counterstaining (blue). Scale bar = 50 μm (original magnification, 400×). (**d**) Quantification of relative LC3 fluorescence intensity (n = 3). Data are presented as mean ± SD. ns, relatively not significant (*p* > 0.05); * *p* < 0.05; ** *p* < 0.01; *** *p* < 0.001.

Previous studies suggest that the cardiovascular protective effects of SalB are intimately linked to the activation of autophagy [25]. Our findings revealed that cold ischemic stress alone (such as in the CS0h group) triggered a basal autophagic response compared to controls, evidenced by increased LC3-II/I ratios, elevated ATG5 and Beclin-1 levels, and the concomitant degradation of p62. Strikingly, SalB supplementation robustly amplified this autophagic flux across all treatment groups (CS4h+SalB, CS8h+SalB, and CS10h+SalB) compared to standard UW preservation. Furthermore, autophagic activity peaked in the CS8h+SalB group, surpassing that of the CS4h control group, with no further significant changes observed at 10 h. Immunofluorescence staining corroborated these findings, confirming enhanced LC3 puncta accumulation in the CS8h+SalB group relative to the CS8h group (Figure 4a–d).

**Figure 5:**
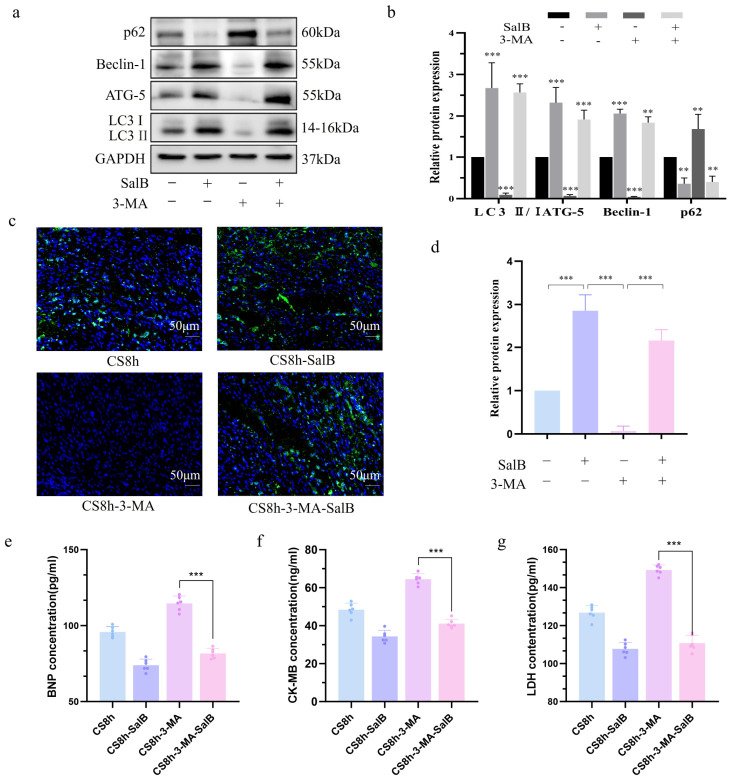
The cardioprotective effect of SalB is partially mediated via counteracting 3-MA-induced autophagic inhibition. (**a**,**b**) Western blot analysis and corresponding quantification of autophagy-related proteins following 3-MA pretreatment (n = 3). (**c**,**d**) Immunofluorescence detection and quantitative analysis of LC3 expression (n = 3). Scale bar = 50 μm (original magnification, 400×). (**e**–**g**) ELISA quantification of myocardial enzyme leakage (BNP, CK-MB, and LDH) demonstrating the functional reversal of 3-MA-induced injury by SalB (n = 6). Data are presented as mean ± SD. Statistical significance was assessed using one-way ANOVA with Tamhane’s T2 test., *p* > 0.05; ** *p* < 0.01; *** *p* < 0.001.

To definitively ascertain whether autophagy mediates the protective effects of SalB, mice were pretreated with the autophagy inhibitor 3-methyladenine (3-MA). As anticipated, while SalB significantly enhanced autophagic flux (CS8h+SalB), the introduction of 3-MA strongly suppressed the expression of autophagy-related proteins (CS8h+3-MA). Interestingly, in the combined treatment group (CS8h+3-MA+SalB), SalB administration partially restored autophagic activity, although it remained below the levels observed in the CS8h+SalB group. Immunofluorescence similarly confirmed an increase in LC3 expression in the CS8h+3-MA+SalB group compared to the 3-MA-only group (Figure 5a–d). Functionally, the abrogation of autophagy by 3-MA exacerbated myocardial injury, resulting in significantly higher levels of myocardial enzymes in the coronary effluent compared to the CS8h+SalB group. Crucially, storage in SalB-supplemented UW solution mitigated this 3-MA-induced exacerbated injury (Figure 5e–g).

## 3. Discussion

Although the University of Wisconsin (UW) solution offers proven efficacy and remains the clinical gold standard for organ preservation [26,27,28,29], its limited safe storage window severely constrains heart transplantation logistics. Salvianolic acid B (SalB), the most abundant water-soluble bioactive compound in *Salvia miltiorrhiza*, is highly soluble in aqueous preservation media, allowing for optimal pharmacological bioavailability. Traditionally recognized for promoting microcirculation and resolving blood stasis, SalB has demonstrated potent protective effects across a spectrum of cardiovascular diseases [30]. Therefore, the present study sought to investigate the cardioprotective efficacy and underlying mechanisms of SalB supplementation (at an established dose of 0.8 mg/mL [31]) during the static cold storage of donor hearts, with the ultimate goal of optimizing the clinical utility of standard UW solution.

Our findings demonstrated that SalB acts as a pleiotropic protective agent during cold ischemia. Specifically, SalB bolstered myocardial antioxidant defenses, as evidenced by preserved SOD activity and suppressed MDA accumulation. Concurrently, SalB exhibited robust anti-inflammatory properties, significantly downregulating the expression of pro-inflammatory cytokines (*IL-1β* and *TNF-α*) relative to time-matched controls. This biochemical preservation translated into functional and structural benefits: SalB supplementation not only markedly reduced the efflux of myocardial injury biomarkers (BNP, CK-MB, and LDH) into the coronary effluent but also preserved the expression of CX43, a critical gap junction protein that is typically degraded during cardiomyocyte injury. Although BNP is traditionally associated with heart failure, its acute release during cold storage reflects ventricular stretch and stress during handling and ischemic edema, serving as an early indicator of myocardial wall injury [32]. Furthermore, histological analysis confirmed that SalB visibly attenuated cold storage-induced pathological alterations, including vacuolar degeneration, interstitial edema, and inflammatory cell infiltration. Crucially, the preservation profile of the 8 h SalB-treated group (CS8h+SalB) was particularly striking. This group exhibited lower myocardial enzyme leakage and higher CX43 expression compared to the 4 h standard storage group (CS4h), while maintaining a comparable antioxidant capacity. Although this compensatory advantage diminished by the 10 h mark, these comparative data compellingly demonstrate that SalB confers profound cardioprotection and successfully extends the viable preservation window.

To elucidate the molecular basis of this cardioprotection, we investigated the interplay between apoptosis and autophagy—two fundamental processes governing cellular survival during ischemic stress. While apoptosis invariably leads to cell death and tissue damage, autophagy serves as an adaptive, pro-survival mechanism that clears damaged organelles and mitigates energy depletion during hypothermic hypoxia. Our mechanistic analyses revealed that SalB treatment orchestrates a distinct pro-survival shift, effectively suppressing myocardial apoptosis while simultaneously amplifying autophagic flux compared to standard UW preservation. To definitively establish causality, we employed the autophagy inhibitor 3-methyladenine (3-MA). The abrogation of autophagic flux by 3-MA significantly reversed the protective benefits of SalB, exacerbating myocardial enzyme leakage and tissue injury. Together, these data substantiate that the SalB-mediated maintenance of cardiac homeostasis during cold storage is heavily dependent on the targeted activation of protective autophagy and the concomitant inhibition of apoptosis. Furthermore, while the use of 3-MA successfully reversed SalB-mediated protection, 3-MA is known to have off-target effects beyond the autophagic pathway. Future studies utilizing genetic approaches (e.g., Atg5 knockdown) are required to establish a definitive causal relationship.

In summary, the supplementation of UW solution with SalB effectively extends the viable static cold ischemia window of donor hearts to 8–10 h. By alleviating oxidative stress, suppressing sterile inflammation, and mitigating apoptosis via the promotion of autophagic flux, SalB significantly optimizes overall preservation outcomes. These preclinical findings highlight SalB as a highly promising, cost-effective pharmacological additive for clinical cardiac graft storage. Nonetheless, a limitation of the current study is its reliance on an ex vivo isolated heart model. Given that prolonged cold storage is a primary risk factor for primary graft dysfunction (PGD) post-transplantation, future in vivo studies utilizing orthotopic heart transplantation models are warranted to verify whether SalB treatment ultimately translates into improved long-term graft survival and functional recovery. Third, this study primarily relied on biochemical and histological evaluations. The lack of functional assessments (e.g., using a Langendorff preparation to measure ventricular pressure and ejection fraction) and in vivo heart transplantation models limits the direct translation of our findings. Future studies employing orthotopic or heterotopic transplantation models are necessary to definitively validate whether SalB improves post-transplant graft survival and contractile function.

## 4. Materials and Methods

### 4.1. Animals

Specific pathogen-free (SPF) male ICR mice (aged 8–10 weeks; weighing 25–30 g) were obtained from the Qinglongshan Experimental Animal Breeding Center. The animals were housed under controlled environmental conditions (temperature: 18–22 °C; humidity: 40–60%) with a standard 12 h light/dark cycle. All animal care and experimental procedures were approved by the Institutional Animal Care and Use Committee (IACUC). The study was approved by the Nanjing University of Chinese Medicine Experimental Animal Ethics Committee (Approval no. 202309A024/2023).

### 4.2. Experimental Design and Grouping

#### 4.2.1. Effects of SalB on Cardiac Preservation Across Different Cold Storage Durations

To evaluate the cardioprotective effects of SalB, mice were allocated into eight experimental groups (n = 12 animals per group) using a random number generator. To ensure data robustness and independent biological replicates for distinct downstream assays, the 12 harvested hearts in each group were predefined and subdivided as follows: six hearts (n = 6) were dedicated to coronary effluent collection and tissue biochemical analyses (e.g., ELISA, RT-qPCR, and oxidative stress assays); three hearts (n = 3) were perfusion-fixed exclusively for histological, TUNEL, and immunofluorescence assessments; and the remaining three hearts (n = 3) were snap-frozen for protein extraction and Western blot analyses. The specific interventions for the eight experimental groups were systematically defined as follows: ① Baseline Control Groups (Control group: Hearts were flushed with 4 °C normal saline and evaluated immediately without subsequent cold storage;

CS0h group: Hearts were flushed with 4 °C standard UW solution and assessed immediately without cold storage). ② Standard UW Cold Storage Groups (CS4h, CS8h, and CS10h groups: Hearts were flushed with 4 °C UW solution and preserved in standard UW solution at 4 °C for 4, 8, and 10 h, respectively). ③ SalB-Supplemented Cold Storage Groups (CS4h+SalB, CS8h+SalB, and CS10h+SalB groups: Hearts were flushed with 4 °C UW solution and preserved at 4 °C for 4, 8, and 10 h, respectively, in UW solution supplemented with 0.8 mg/mL SalB). The concentration of SalB (0.8 mg/mL) was selected based on our preliminary dose–response experiments and previous literature [33], which demonstrated optimal cytoprotective efficacy without inducing cytotoxicity.

#### 4.2.2. Role of Autophagy in SalB-Mediated Cardioprotection

To investigate the involvement of autophagy, a separate cohort of mice was randomly allocated into four groups (n = 4 per group). All hearts in this cohort were subjected to 8 h of cold storage at 4 °C. The treatment groups were as follows: (1) CS8h (preservation in standard UW solution); (2) CS8h+SalB (preservation in UW solution containing 0.8 mg/mL SalB); (3) CS8h+3-MA (intraperitoneal injection of 2 mg/kg 3-methyladenine (3-MA) prior to anesthesia, followed by preservation in UW solution); and (4) CS8h+3-MA+SalB (intraperitoneal injection of 3-MA prior to anesthesia, followed by preservation in UW solution supplemented with SalB).

### 4.3. Perfusion of Isolated Mouse Hearts

The ex vivo cardiac perfusion model utilized retrograde aortic perfusion. Briefly, perfusate delivered into the aorta creates retrograde flow, which closes the aortic valve, thereby directing the solution into the coronary arteries from the aortic root. The perfusate then circulates through the myocardium, enters the coronary sinus, and ultimately exits via the right atrium, pulmonary artery, and vena cava.

**Figure 6:**
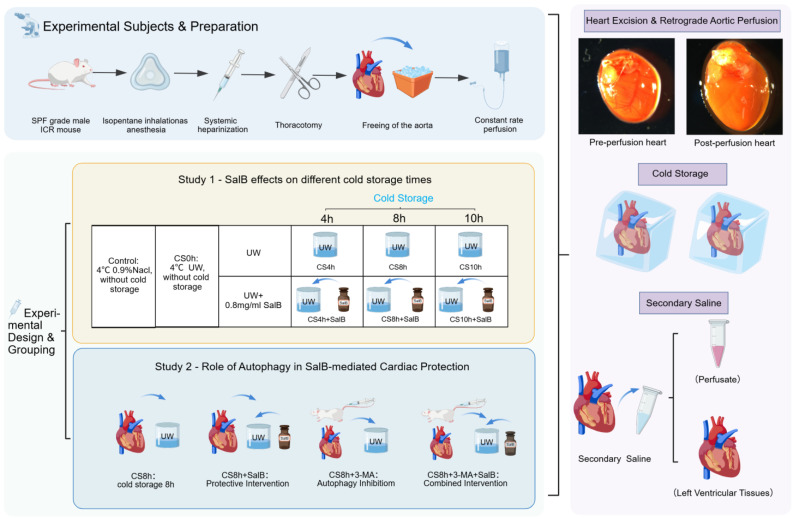
Schematic illustration of the experimental design, subject preparation, and group allocation strategy. (Created with BioGDP.com [34]).

Mice were anesthetized via continuous inhalation of isoflurane and placed on a surgical stage. Following systemic heparinization, a median thoracotomy was performed. Topical cooling was applied by placing ice into the thoracic cavity, and the aorta was isolated. The hearts were rapidly excised and perfused with the designated preservation solutions according to the experimental groups. Following the cold storage period, a secondary perfusion with normal saline was performed. The coronary effluent (perfusate) and cardiac tissues were subsequently collected for downstream analyses (Figure 6).

### 4.4. Detection of Myocardial Enzymes

The levels of B-type natriuretic peptide (BNP), creatine kinase-MB (CK-MB), and lactate dehydrogenase (LDH) in the coronary effluent were quantified using commercial enzyme-linked immunosorbent assay (ELISA) kits (Abmart, Shanghai, China) in accordance with the manufacturer’s instructions. According to the manufacturer’s specifications, the detection ranges for the BNP, CK-MB, and LDH assays were 12.35–1000 pg/mL, 0.78–100 ng/mL, and 7.8–500 ng/mL, respectively. The analytical sensitivities for these assays were 4.84 pg/mL, 0.32 ng/mL, and 3.1 ng/mL, respectively. The optical density (OD) was measured at 440 nm using a microplate reader (Allsheng Instruments, Hangzhou, China), and the values were converted to enzyme concentrations based on standard curves.

### 4.5. Antioxidant Capacity Detection

Left ventricular myocardial tissues were homogenized in ice-cold buffer and centrifuged at 3000 rpm for 10 min at 4 °C, after which the supernatants were collected. Superoxide dismutase (SOD) activity and malondialdehyde (MDA) content were assayed using commercial kits (Nanjing Jiancheng Institute of Bioengineering, Nanjing, China) according to the manufacturer’s protocols. The OD values were read at 450 nm and 532 nm, respectively, using a microplate reader (Allsheng Instruments), and the data were calculated to reflect tissue antioxidant capacity.

### 4.6. Reverse Transcription-Quantitative Polymerase Chain Reaction (RT-qPCR)

Total RNA was extracted from myocardial tissues using TRIzol reagent (Invitrogen, Carlsbad, CA, USA). cDNA synthesis and subsequent qPCR were performed using a SYBR Green Master Mix (Yeasen, Shanghai, China) according to the manufacturer’s protocol. Relative mRNA expression levels were calculated applying the 2^−ΔΔCt^ method. The primer sequences (synthesized by Sangon Biotech, Shanghai, China) were as follows: *interleukin (IL)-1β* (forward: 5′-TCGCTCAGGGTCACAAGAAA-3′; reverse: 5′-CATCAGAGGCAAGGAGGAAAAC-3′); and *tumor necrosis factor-α (TNF-α)* (forward: 5′-ACGTGGAACTGGCAGAAGAG-3′; reverse: 5′-CTCCTCCACTTGGTGGTTTG-3′).

### 4.7. Hematoxylin and Eosin (H&E) Staining

Left ventricular tissues were fixed in 4% paraformaldehyde and embedded in paraffin blocks. Following deparaffinization and rehydration, 4-μm-thick tissue sections were stained with hematoxylin and eosin (H&E). The stained sections were then observed and imaged using a light microscope (Leica, Wetzlar, Germany).

### 4.8. Western Blotting

Myocardial tissues were lysed in radioimmunoprecipitation assay (RIPA) buffer (Solarbio, Beijing, China) to extract total protein. Equivalent amounts of protein were separated by sodium dodecyl sulfate-polyacrylamide gel electrophoresis (SDS-PAGE) and transferred onto polyvinylidene difluoride (PVDF) membranes. After blocking, the membranes were incubated overnight (16 h) at 4 °C with the following primary antibodies: Bax (1:5000, 50599-2-Ig), Bcl-2 (1:5000, 68103-1-Ig), ATG5 (1:5000, 10181-2-AP), p62 (1:10000, 18420-1-AP), and CX43 (1:5000, 26980-1-AP) (all from Proteintech, Wuhan, China); as well as GAPDH (1:1000, P30008), Cleaved Caspase-3 (1:1000, TA7022), LC3 (1:1000, T55992), and Beclin-1 (1:1000, T55092) (all from Abmart, Shanghai, China). Subsequently, the membranes were incubated with a horseradish peroxidase (HRP)-conjugated goat anti-rabbit or anti-mouse IgG secondary antibody (1:5000; Cell Signaling Technology, Beverly, MA, USA) for 2 h at room temperature. Immunoreactive bands were visualized using an enhanced chemiluminescence (ECL) detection system.

### 4.9. Terminal Deoxynucleotidyl Transferase dUTP Nick End Labeling (TUNEL) Staining

Left ventricular tissues were fixed in 4% paraformaldehyde, embedded in paraffin, and sectioned at an 8-μm thickness. Following deparaffinization and rehydration, the sections were subjected to TUNEL staining to detect apoptotic cells in accordance with the manufacturer’s protocol. Nuclei were subsequently counterstained with hematoxylin. Finally, the sections were dehydrated, mounted, and photographed under a light microscope (Leica).

### 4.10. Immunofluorescence

Tissue sections were fixed with 4% paraformaldehyde for 30 min, permeabilized with 0.25% Triton X-100 for 15 min, and blocked with 1% bovine serum albumin (BSA) for 1 h at room temperature. The sections were then incubated with a primary antibody against LC3 (1:100, T55992, rabbit; Abmart) overnight at 4 °C. Following PBS washes, the sections were incubated with a fluorophore-conjugated goat anti-rabbit IgG (H + L) secondary antibody (1:500, SA00013-2; Proteintech) for 1 h at 37 °C. Cell nuclei were counterstained with 4′,6-diamidino-2-phenylindole (DAPI). Images were acquired using a fluorescence or laser-scanning confocal microscope (Leica). The nuclear and cytoplasmic fluorescence intensities were quantified using the Columbus image analysis server and the Operetta CLS High-Content Analysis System (PerkinElmer, Waltham, MA, USA).

### 4.11. Statistical Analysis

Mice were randomly assigned to experimental groups using a random number generator. Furthermore, researchers performing histological scoring, image analysis, and Western blot quantification were blinded to the group allocations.

All data are presented as the mean ± standard deviation (SD). Statistical significance between two groups was evaluated using the independent Student’s *t*-test, whereas comparisons among multiple groups were performed using one-way or two-way analysis of variance (ANOVA), followed by appropriate post hoc tests. A *p*-value of < 0.05 was considered statistically significant. Statistical analyses were conducted using [e.g., GraphPad Prism 9.0 or SPSS 26.0].

## 5. Conclusions

In conclusion, the present study demonstrates that supplementing standard University of Wisconsin (UW) solution with Salvianolic acid B (SalB) significantly mitigates cold ischemic injury in isolated mouse hearts. This robust cardioprotection is achieved through the attenuation of oxidative stress, sterile inflammation, and cardiomyocyte apoptosis, coupled with the targeted enhancement of autophagic flux. Notably, SalB effectively extends the viable cold preservation window to 8–10 h. The abrogation of these protective benefits by the autophagy inhibitor 3-MA confirms that the modulation of autophagy is a pivotal mechanistic pathway underlying the efficacy of SalB. Consequently, SalB emerges as a highly promising, clinically translatable pharmacological additive for optimizing cardiac graft preservation. Nevertheless, future in vivo orthotopic transplantation studies are imperative to validate its capacity to prevent primary graft dysfunction (PGD) and improve long-term post-transplant survival.

## Data Availability

The original contributions presented in this study are included in the article. Further inquiries can be directed to the corresponding author.

## References

[1] Rowart P., Erpicum P., Detry O., Weekers L., Grégoire C., Lechanteur C., Briquet A., Beguin Y., Krzesinski J.-M., Jouret F. (2015). Mesenchymal stromal cell therapy in ischemia/reperfusion injury. J. Immunol. Res..

[2] Li J., Peng Q., Yang R., Li K., Zhu P., Zhu Y., Zhou P., Szabó G., Zheng S. (2021). Application of mesenchymal stem cells during machine perfusion: An emerging novel strategy for organ preservation. Front. Immunol..

[3] Hosgood S.A., Brown R.J., Nicholson M.L. (2021). Advances in Kidney Preservation Techniques and Their Application in Clinical Practice. Transplantation.

[4] Tingle S.J., Thompson E.R., Figueiredo R.S., Moir J.A., Goodfellow M., Talbot D., Wilson C.H. (2024). Normothermic and hypothermic machine perfusion preservation versus static cold storage for deceased donor kidney transplantation. Cochrane Database Syst. Rev..

[5] Paez J.R., White R.E., Dunn K., Gopagani L., Pham S., Pahinka D., Chivukula V.K. (2025). Investigating cardiac temperature during heart transplantation using the static cold storage paradigm. Transplantation.

[6] McAnulty J.F. (2010). Hypothermic organ preservation by static storage methods: Current status and a view to the future. Cryobiology.

[7] Verstraeten L., Den Abt R., Ghesquière B., Jochmans I. (2023). Current Insights into the Metabolome during Hypothermic Kidney Perfusion-A Scoping Review. J. Clin. Med..

[8] Stewart Z.A. (2015). UW solution: Still the “gold standard” for liver transplantation. Am. J. Transplant..

[9] Wang X., Yin X., Gao Y., Liu W., Jiang D., Zhang L., Zhang C. (2026). Machine Perfusion Ameliorates Nonanastomotic Strictures After Liver Transplantation: A Systematic Review and Meta-analysis of Randomized Controlled Trials. Clin. Transl. Gastroenterol..

[10] Petrenko A., Carnevale M., Somov A., Osorio J., Rodríguez J., Guibert E., Fuller B., Froghi F. (2019). Organ preservation into the 2020s: The era of dynamic intervention. Transfus. Med. Hemother..

[11] Zhong Y.J., Wang C. (2022). Research progress on the application and mechanism of traditional Chinese medicine in organ preservation solution. J. Med. Res..

[12] Yang S., Chen H., Su W., Luo Y., Liao J., Wang Y., Xiong L., Zhang C., Li F., Chen Z.-S. (2023). Protective effects of Salvianic acid A against multiple-organ ischemia-reperfusion injury: A review. Front. Pharmacol..

[13] Wang H., Zhang H., Lai C., Chen Y. (2025). Salvianolic acid B ameliorates lipopolysaccharide-induced inflammatory liver injury via deacetylation of NF-κB RelA. Food Biosci..

[14] Wang Y., Wu J., Lai Y., Li D., Huang Y., Wang X., Zhou Y., Xiao H., Meng X., Qian J. (2026). Multitargeted synergistic mechanisms of Salvia miltiorrhiza and its bioactive compounds: A review. J. Ethnopharmacol..

[15] Han J., Li Q., Sun K., Pan C., Liu J., Huang P., Feng J., Liu Y., Meininger G.A. (2024). Natural Products Improve Organ Microcirculation Dysfunction Following Ischemia/Reperfusion- and Lipopolysaccharide-Induced Disturbances: Mechanistic and Therapeutic Views. J. Pharm. Anal..

[16] Li G. (2021). Salvia polyphenolate reduces the intestinal ischemia-reperfusion injury by inhibiting endoplasmic reticulum stress. Tianjin Univ. Tradit. Chin. Med..

[17] Liu Y., Ao X., Hao W., Wang H., Li L., Wang S., Li J., Liu J., Zhou X., Li Z. (2026). Research Progress on Salvia miltiorrhiza Bioactive Components Regulating P-Selectin for Microcirculatory Improvement: Potential Implications in Acute Pancreatitis. Mediat. Inflamm..

[18] Zhou S., Wang L., Wang W., Zheng H. (2025). Effect and mechanism of Danshen injection in improving intestinal mucosal barrier function in rats with adhesive intestinal obstruction. Chin. J. Gen. Surg..

[19] Ren Z.X., Zhang Y.Y. (2019). Research progress on chemical constituents and pharmacological effects of salvianolic acid B. Shandong Chem. Ind..

[20] Zhang H. (2021). Analysis of clinical significance of myocardial enzyme profiling in patients with acute myocardial infarction. Mod. Health.

[21] Sun S.W. (2021). Analysis of BNP, cTnI and myocardial enzyme index detection in the clinical diagnosis of patients with acute heart failure. Heilongjiang J. Tradit. Chin. Med..

[22] Li X.X., Shan Y.H., Luo S.Y., Wang L.Y. (2020). Correlation between the expression of proinflammatory factor and anti-inflammatory factor in peripheral blood of patients with sepsis and myocardial injury. Chin. J. Pract. Med..

[23] Prabhu S.D., Frangogiannis N.G. (2016). The biological basis for cardiac repair after myocardial infarction: From inflammation to fibrosis. Circ. Res..

[24] Epifantseva I., Shaw R.M. (2018). Intracellular trafficking pathways of Cx43 gap junction channels. Biochim. Biophys. Acta Biomembr..

[25] Lin P.L., Wu M., Yang F., Xu L., Huang D., Qiu F.R., Ye Z.Y. (2022). Mechanism of salvianolic acid B activation of autophagy to improve calcification of vascular smooth muscle cells induced by high phosphorus. Chin. J. Hosp. Pharm..

[26] George T.J., Arnaoutakis G.J., Beaty C.A., Shah A.S., Conte J.V., Halushka M.K. (2012). A novel method of measuring cardiac preservation injury demonstrates University of Wisconsin solution is associated with less ischemic necrosis than Celsior in early cardiac allograft biopsy specimens. J. Heart Lung Transplant..

[27] Hassanzadeh P., Lam A., Wagner M.J., Conway J., Freed D.H. (2026). Advancements in Donor Heart Preservation Methods: A Review of Approaches. Can. J. Cardiol..

[28] George T.J., Arnaoutakis G.J., Baumgartner W.A., Shah A.S., Conte J.V. (2011). Organ storage with University of Wisconsin solution is associated with improved outcomes after orthotopic heart transplantation. J. Heart Lung Transplant..

[29] Latchana N., Peck J.R., Whitson B., Black S.M. (2014). Preservation solutions for cardiac and pulmonary donor grafts: A review of the current literature. J. Thorac. Dis..

[30] Lin C., Liu Z.G., Qian X., Yao Y., Xu B., Bie H.M. (2015). Advances in pharmacological effects of salvianolic acid B on cardiovascular diseases. Chin. Pharmacol. Bull..

[31] Xu H., Sun M., He Y.J., Sun J., Zhang J., Chen J.Y. (2014). Protective effect of salvianolic acid B on isolated donor lung in rats. Chin. J. Organ. Transplant..

[32] Li J., Yin F.F., Hou Y.L. (2013). Early diagnosis of rats with acute myocardial infarction by measurement of brain natriuretic peptide. Exp. Ther. Med..

[33] Sun M., Li H., Xu H., Chen J.Y., Sun J., Ji Y. (2015). Protective Effect and Mechanism of Raffinose-low Potassium Dextran Added Salvianolic Acid B on Isolated Lung in Rats. Prog. Mod. Biomed..

[34] Jiang S., Li H., Zhang L., Mu W., Zhang Y., Chen T., Wu J., Tang H., Zheng S., Liu Y. (2025). Generic Diagramming Platform (GDP): A comprehensive database of high-quality biomedical graphics. Nucleic Acids Res..

